# The effects of embryo culture media on the birthweight of singletons via fresh or frozen-thawed embryo transfer: a large-scale retrospective study

**DOI:** 10.1186/s12884-016-1077-7

**Published:** 2016-09-19

**Authors:** Fang Gu, Mingfen Deng, Jun Gao, Zilian Wang, Chenhui Ding, Yanwen Xu, Canquan Zhou

**Affiliations:** 1 Department of Obstetrics and Gynecology, Reproductive Medical Center, The First Affiliated Hospital of SunYat-senUniversity, No 58 Zhongshan Road 2, Yuexiu District Guangzhou, 510080 China; 2 Key Laboratory of Reproductive Medicine of Guangdong Province, Guangzhou, China; 3 Department of Obstetrics and Gynecology, The First Affiliated hospital of SunYat-senUniversity, No 58 Zhongshan Road 2, Yuexiu District Guangzhou, 510080 China

**Keywords:** IVF, Birthweight, Embryo culture medium, Singleton, Vanishing twin

## Abstract

**Background:**

Embryo culture media used for IVF treatment might affect fetal growth and thus birthweight of the newborns.

**Methods:**

A retrospective study was conducted in South China using data from 2370 singleton neonates born after IVF/ICSI between 2009 and 2012. Two culture media, i.e., either Vitrolife or SAGE were used as embryo culture media during the study period. Neonates’ birthweights were compared between the two embryo culture media groups.

**Results:**

Among the 2370 singletons, 1755 cases came from fresh cleavage embryo transfer while 615 were from frozen-thawed cleavage embryo transfer. Within the fresh embryo transfer newborns, no statistical difference was observed in either birthweight (mean ± SD: 3196.0 ± 468.9 versus 3168.4 ± 462.0g, *p* > 0.05) or adjusted birthweight controlled for gestational age and gender (z-score mean ± SD: 0.11 ± 1.02 versus 0.11 ± 0.99 g, *P* > 0.05) between the Vitrolife (*n* = 419) and the SAGE group (*n* = 1336). Likewise within frozen embryo transfer neotates, no statistical difference of the birthweight (3300.6 ± 441.3 vs.3256.0 ± 466.7 g, *P* > 0.05) and adjusted birthweight (0.30 ± 0.99 g versus 0.29 ± 0.97 g, *P* > 0.05) was found between the Vitrolife (*n* = 202) and the SAGE group (*n* = 413). The sex ratio [OR1.17, 95 % CI (0.94–1.46)/OR1.1, 95 % CI (0.78–1.54)], rate of small for gestational age [OR1.14, 95 % CI (0.82–1.59)/OR1.06, 95 % CI (0.56–2.02)] and large for gestational age [OR1.07, 95 % CI (0.64–1.76)/OR0.98, 95 % CI (0.47–2.02)] in fresh and frozen-thawed subgourps are all comparable respectively between the two culture media. No group differences were found in the rate of low birthweight and macosomia. Multiple linear regression analysis demonstrated that maternal weight, gestational age, frozen-thawed embryo transfer and infant gender were significantly related to neonatal birthweight (*P* < 0.001).

**Conclusions:**

It appears that embryos cultured in SAGE or Vitrolife media after fresh or frozen-thawed cleavage embryo transfer did not affect neonate’s birthweight.

## Background

The Assisted Reproductive Technology (ART) is thought to be associated with an increased risk of adverse perinatal outcomes such as preterm birth, low birth weight, fetal malformation and being born small for gestational age (SGA) or large for gestational age (LGA) [1–5]. Despite the high prevalence of twins and triplets associated with ART which contributes to the increased risk of these adverse outcomes, singletons born after ART also performed worse than their naturally conceived peers [6]. Nowadays, the aspects of ART itself believed to affect perinatal outcomes are ovarian stimulation, laboratory procedures and the culture media [7].

Whether embryo culture media affect fetal growth and birthweight remain inconclusive. Several studies showed that culture media used during IVF treatment could influence intrauterine growth and birthweight of newborns [8, 9]. In contrast, other research did not confirm such an adverse effect [10–12].

Vitrolife and SAGE medium are the two commonly used embryo culture media in China. They are both sequential culture media for embryo culture with similar components. SAGE is produced in the United States (US) and mainly used in patients in US and Asia, while Vitrolife is more widely used in Europe. Thus it would be interesting to compare the perinatal outcome of these two culture media in the same population under the same laboratory condition. As such, a retrospective study was conducted to investigate the effect of these two culture media, Vitrolife and SAGE, on the birthweight of singleton newborns in a large population conceived from fresh and frozen-thawed embryo transfer.

## Methods

### Study design and study population

This was a retrospective study. Subjects were singletons born alive over 28 weeks of gestation who underwent IVF/ICSI cycles with cleavage embryo transfer between June 2009 and October 2012 in the reproductive medical center of the First Affiliated Hospital of SunYat-sen University. Women applying for blastocyst transfer, preimplantation genetic diagnosis were excluded from the study. The research protocol was approved by Ethic Review Committee of the First Affiliated Hospital of SunYat-sen University and informed consents were obtained prior to data collection. Research nurses contact mothers to obtain information on perinatal outcomes including birthweight, gestational age and gender. Pregnancies lost to follow-up were excluded from data analysis.

### Stimulation regimens

Women underwent controlled ovarian hyperstimulation with a GnRH agonist and antagonist protocol. Ovarian follicle development was monitored based on serum estradiol (E_2_) levels and transvaginal ultrasonographic measurements. When at least three follicles reached a mean diameter of 18 mm, 250 μg recombinant hCG (Serono, Aubonne, Switzerland) was administered 36 h before ultrasonography-guided oocyte retrieval. Luteal support was initiated on the day after oocyte retrieval using 40 mg of progesterone.

### Laboratory protocols

Two culture media were used: G5™ (Vitrolife, Gottenburg, Sweden) and Quinn’s advantage medium (SAGE, CA, USA) for embryo culture. The corresponding sera supplemented the media: HSA solution™ (Vitrolife) and Quinn’s advantage SPS (SAGE). Oocytes were placed in either SAGE or Vitrolife after oocyte retrieval. Then IVF/ICSI were performed according to the laboratory’s routine insemination procedures. The presence of two pronuclei was observed 16–18 h after insemination or injection, and the zygotes were then cultured in 25 μl pre-equilibrated cleavage medium droplets (G1and Quinn’s advantage cleavage medium). The embryos were cultured in incubators at 37.0 °C under 6 % CO_2_ in Vitrolife group and 5 %CO_2_ in SAGE group strictly following the product instructions. The morphology of embryos was evaluated 68–72 h after insemination with respect to cell number, fragmentation and symmetry.

Embryo quality assessment protocol was based on the standard of Istanbul consensus workshop on embryo assessment [13]. The scoring format was cell number, grade and fragmentation. “Good quality” Day-3 embryos were defined as 7–9 equally sized mononucleated blastomeres, with < 10 % fragmentation.

### Embroy transfer protocols

On day 3 after oocyte retrieval, embryo transfer was provided and the number of transfer embryos was determined based on patient’s age, number of IVF cycles and embryo quality. Normally, we transferred two Day 3 embryos in patients under 35 years old in their first cycle and transferred three Day3 embryos in other cases. Surplus good quality embryos were cryopreserved through slow-freezing or vitrification.

In frozen embryo transfer (FET) cycles, endometrium preparation protocols including natural cycle and hormone replacement treatment (HRT) cycle were administrated according to the characteristics of patients. The natural cycle preparation protocol included cycle evaluation using ultrasound monitoring of follicular size and endometrial thickness. The day of ovulation was determined by the disappearance of the leading follicle combined with decline of E_2_ and elevation of progesterone level. Embryos were transferred on Day 3 after ovulation. HRT cycles were performed to patients who did not have regular ovulation. Participants started oral administration of 4 mg/d E_2_valerate (Progynova, Schering Health Care) for endometrium preparation. An ultrasound assessment was done 12 to 14 days later to assess the uterus as ready for the ET procedure when the endometrium thickness exceeded 8 mm. If not adequate, endometrial priming continued with step-up dose of E_2_ and monitoring scans were undertaken to confirm further endometrial thickening. Participants commenced luteal support via 40 mg progesterone injection for three days before ET.

### Criteria for data collection

Birthweight comparison was our major goal. As gestational age and gender are crucial factors for fetal birthweight [14], we not only compared the absolute birthweight but also the birthweight adjusted for gestational age and gender, which was known as the z-score. The z-score of each individual case was calculated using the following formula: “the weight of the individual child minus the median weight of a reference population of children born at the same gestational age and of the same gender divided by the standard deviation (at the same gestational age and the same gender)” [15, 16]. The reference population represented the latest available birthweight data for the same province in China [17]. We defined low birthweight (LBW) and macrosomia according to the international description [18]. Small for gestational age (SGA) and large for gestational age (LGA) were defined based on the standards referenced in the book of Chinese Perinatology [19].

### Statistical analysis

All statistical analyses were performed with SPSS 17.0 software. The basic characteristics of the patients were compared using Student’s t-tests (continuous variables) and categorical variables were evaluated with *X*^2^tests. Multiple linear regression analyses were used to evaluate the association between culture media and birthweight, while controlling for the effects of possible confounding factors, such as fertilization methods (IVF or ICSI), fresh or frozen-thawed embryo transfer, maternal age, maternal weight, gestational age and infant gender.

## Results

### Patient and cycle characteristics

A total of 2370 newborns were studied. Among these, 1755 were babies delivered from fresh embryo transfer with 419 cultured in Vitrolife and 1336 in SAGE; while 615 cases were delivered from frozen cleavage embryo transfer group, with 202 embryos cultured in Vitrolife and 413 cultured in SAGE. Each couple contributed only one pregnancy to this study.

The maternal characteristics are shown in Tables 1 and 2. All patients were Chinese non-smoking women. No significant differences were found between these two media in patients’ characteristics, except maternal age in the fresh embryo transfer group (30.8 ± 4.1 years versus 31.5 ± 4.2 years, *p* < 0.05). GnRH agonist long protocol dominated our ovarian stimulation protocol with 97.3 % in Vitrolife group and 96.4 % in Sage group, while the percentages of GnRH antagonist were only 2.6 vs 3.3 %, *p* > 0.05. The average numbers of oocytes collected were 12.4 ± 5.8 and 12.07 ± 6.1 respectively. IVF/ICSI fertilization rates were comparable,74.0/81.7 % in Vitrolife versus 75.6/81.8 % in SAGE with similar numbers of embryos per patient (5.2 ± 0.1 verse 5.0 ± 0.2, *p* > 0.05).

**Table 1:** Patients’ characteristics and neonatal outcome after fresh cleavage-stage embryo transfer

	Vitrolife (*n* = 419)	SAGE (*n* = 1336)	*p*
Maternal age (years)	30.84 ± 4.05	31.47 ± 4.17	0.007
Maternal weight (Kg)	52.63 ± 6.73	52.10 ± 6.79	0.22
Body Mass Index (kg/m^2^)	20.77 ± 2.79	20.73 ± 3.18	0.81
Nulliparous	388 (92.6)	1244 (93.1)	0.74
Gestational age (weeks)	38.47 ± 1.43	38.32 ± 1.50	0.07
Birthweight (g)	3196.0 ± 468.9	3168.4 ± 462.0	0.29
Birthweight z-score	0.11 ± 1.02	0.11 ± 0.99	0.10
Low Birthweight (<2500 g)	21 (5.0)	68 (5.1)	1.00
Macrosomia (>4000 g)	23 (5.5)	49 (3.7)	0.12
Boys	236 (56.3)	700 (52.3)	0.16
Preterm birth	27 (6.4)	90 (6.7)	0.91
Small for gestational age (SGA)	54 (12.9)	153 (11.4)	0.44
Large for gestational age (LGA)	21 (5.0)	63 (4.7)	0.80

Data are presented as number (%) or mean ± SD

**Table 2:** Patients’ characteristics and neonatal outcome after frozen-thawed cleavage-stage embryo transfer

	Vitrolife (*n* = 202)	SAGE (*n* = 413)	*p*
Maternal age (years)	30.07 ± 3.79	30.64 ± 3.86	0.08
Maternal weight (Kg)	52.58 ± 7.56	52.24 ± 7.00	0.57
Body Mass Index (kg/m^2^)	20.35 ± 3.66	20.62 ± 2.82	0.32
Nulliparous	186 (92.1)	383 (92.7)	0.74
Gestational age (weeks)	38.68 ± 1.34	38.39 ± 1.59	0.03
Birthweight (g)	3300.6 ± 441.3	3256.0 ± 466.7	0.27
Birthweight z-score	0.30 ± 0.99	0.29 ± 0.97	0.96
Low Birthweight (<2500 g)	7 (3.6)	15 (3.7)	1.00
Macrosomia (>4000 g)	12 (6.2)	16 (4.0)	0.23
Boys	105 (52)	205 (49.6)	0.61
Preterm birth	8 (4.3)	23 (6.0)	0.38
Small for gestational age (SGA)	15 (8.2)	29 (7.6)	0.80
Large for gestational age (LGA)	11 (6.0)	23 (6.1)	0.33

Data are presented as number (%) or mean ± SD

### Perinatal outcome

The birth outcomes are shown in Tables 1 and 2. For fresh cleavage embryo transfer, the average weight of newborns was 3196.0 ± 468.9 g in Vitrolife group and 3168.4 ± 462.0 g in SAGE. For cryopreserved cleavage-stage embryo transfer, the mean birthweight was 3300.6 ± 441.3 g and 3256.0 ± 466.7 g in Vitrolife and SAGE, respectively.

Z-score values were used to further explore birthweight differences while adjusting for infant gender and gestational age. Similar to absolute birthweight, there was no significant association between the z-score values and the type of embryo culture medium.

Of the pregnancies resulting in a singleton live birth, no differences were found with respect to the rate of SGA and LGA, as well as the LBW and macrosomia (Tables 1 and 2). Gestational age was found to be slightly but significantly higher in frozen cleavage embryos cultured in Vitrolife than in SAGE media (38.68 ± 1.34 weeks versus 38.39 ± 1.59 weeks, *p* < 0.05).

Finally, multiple linear regression was used to determine the relationship between culture medium and birthweight, gestational age, infant gender, type of culture media, maternal age, maternal weight, fertilization method, fresh or frozen-thawed embryo transfer. Maternal weight, gestational age, male sex and frozen-thawed embryo transfer were found to correlate with birthweight positively (Table 3).

**Table 3:** Results of multiple regression analysis between birthweight and confounding factors among live born singletons

	β	t	*p*
Maternal age (per year)	−0.008	−0.45	0.65
Maternal weight (per kg)	0.211	11.77	<0.001
Frozen thawed embryo (versus fresh embryo)	0.079	4.25	<0.001
ICSI (versus IVF)	0.009	0.48	0.63
SAGE (versus Vitrolife)	−0.004	−0.24	0.81
Gestational age	0.490	27.38	<0.001
Gender (male versus female)	0.110	6.33	<0.001

β is the regression coefficient

### Survivors of a vanished co-twin

The 2370 singleton newborns included 239 survivors of a vanished co-twin,10 survivors of a vanished triplet and 2121 primary singletons. Among the 239 survivors of a vanishing co-twin, 209 babies were from fresh embryo transfer and 30 were born after frozen embryo transfer. The proportion of survivors in Vitrolife and in SAGE were similar at 12.2 versus 11.8 % in fresh embryo transfer. While in frozen embryo cohort, singleton survivors cultured in Vitrolife and in SAGE were in the proportion of 3.4 versus 5.5 %, which makes no statistical difference. Furthermore, considering the gestational age at the vanishing time, the proportions of early, intermediate and late survivors from each culture media were also comparable (Table 4).

**Table 4:** Proportion of singletons originated from twin/triplet gestation and the gestational age at the time of vanishing

	Fresh			Frozen-thawed		
	Vitrolife	SAGE	*p*	Vitrolife	SAGE	*p*
Survivors from twins	51 (12.2)	158 (11.8)	0.81	7 (6.2)	23 (6.0)	0.26
Survivors from triplets	2 (0.5)	5 (0.4)	0.68	2 (1.0)	1 (0.2)	0.25
Early pregnancy loss	28 (6.6)	66 (4.9)	0.17	2 (1.0)	9 (2.4)	0.5
Intermediate pregnancy loss	22 (5.3)	86 (6.4)	0.42	5 (2.2)	14 (3.4)	0.06
Late pregnancy loss	1 (0.2)	6 (0.4)	1.0	0	0	N/A

Data are presented as number (%)

N/A: Non-applicable

## Discussion

The main finding of this retrospective study was that the type of culture medium did not significantly affect the birthweight in singletons after either fresh or frozen-thawed cleavage-stage embryo transfer. The sex ratio, rate of SGA and LGA as well as the rate of LBW and macrosomia were all comparable between the two culture media.

Culture medium is essential for embryo development in IVF. However, the types and concentrations of nutrients contained in the culture medium vary amongst different brands [20]. Nowadays, seven types of culture media are commercially available [21]. Two are single-step culture media which include IVF Online and Irvine Scientific and five are sequential culture media including Cook Medical, In Vitro Care, Origio, SAGE and Vitrolife.

Increasing evidence indicated that embryo culture may adversely affect the developmental potential and overall health of the embryo. In animal experiments, embryo culture in commercial media systems were proven to result in imprinted methylation loss compared to in vivo-derived embryos [22]. Blastocyst and fetal development rates across different culture media showed profound variation in mouse zygotes [23]. Yet the results of mouse embryo responding to human media cannot be transferable directly to human development due to the physiological differences between oocytes from the two distinct species.

As a result, many researchers have started to study the influence of culture media on neonatal birthweight. Dumoulin et al. [24] found that singletons conceived after IVF with fresh cleavage Day 2/3 embryo transfer cultured in Vitrolife medium were heavier than those conceived after culturing in Cook medium. He further reinforced his conclusion in a small size cohort study of both fresh and frozen embryo transfer [18, 24]. Likewise, Eskildet al. [25] found that the birthweight as well as the placental weight differed between different culture media. However, recently studies comparing culture media found no significant differences in mean birthweight between singletons cultured in different media such as Global/Vitrolife/G1.3media;G1.3/Global/G1.5media; Medicult/Cook/Vitrolifemedia; Medicult/Cook and Vitrolife/Global/SAGE media [10–12, 26, 27].

The major differences between Vitrolife and SAGE are the protein source and the ratio of lactate to pyruvate (L:P). Both HSA (Human serum albumin) and SSS (Synthetic serum supplement) were used in Vitrolife while only SSS was used in SAGE. Vitrolife has higher concentration of lactate and higher ratio of lactate to pyruvate, both of which are primary energy substrates for early embryos. Further, SAGE were found additional electrolytes and metals such as aluminum and manganese which were not listed as media components [21]. More surprisingly, most commercial embryo culture media including Vitrolife and Cook medical were proved containing a large variety of non-declared protein which could potentially influence embryonic development, gestational age and birthweight [28].

SAGE is mainly used in reproductive centers of the US and Asia. The singleton birthweight was reported vary between 3020 and 3341 g in frozen cleavage embryo transfer and 2940 to 3350 g after transferring fresh embryo cultured in SAGE [12, 29]. Vitrolife is, in contrast, more widely used in Europe and the reported birthweight varied between 2942 and 3453 g after Day 3 fresh embryo transfer and was reported as 3394 g in frozen-thawed group [8, 9].

It would therefore be interesting to compare the perinatal outcome of these two culture media in the same population as well as the same laboratory condition. To our knowledge, there has been only one study published comparing the effects of SAGE and Vitrolife media on the birthweight of neonates after fresh cleavage embryo transfer. It was reported that no difference was found between the mean birthweight, with 3246.10 ± 220.6 g in 792 Vitrolife babies and 3293.9 ± 262.6 g in 235 SAGE newborns [11]. Our results were consistent with them but with a lower mean birthweight. The birthweight was 3196.0 ± 468.9 g on average in the Vitrolife group and 3168.4 ± 462.0 g in the SAGE group. This small difference in birthweight might be due to the different population and life styles between the North and South of China. However, the previous study only compared the absolute birthweight without adjusting for gestational age and gender and lacked the associated data on frozen-thawed embryo transfer.

Okenet al. [14] found that gestational age and gender of the infant were the factors with the most influences on birthweight in singleton deliveries. Therefore accordingly, we used the adjusted z-score [25, 30] to give a more accurate picture for birthweight comparisons. Furthermore, most of the previous studies have only focused on the birth outcomes from fresh Day2/3 embryo transfer and few of them had data from the frozen-thawed embryo transfer. Our results were in agreement with most of the previous researches but gave more information on the effects of culture media by comparing them in both fresh and frozen-thawed cleavage embryo transfer groups.

Consistent with other studies, our multiple regression analyses showed a significant positive correlation between maternal weight and birthweight as well as gestational age and birthweight [11]. Male sex and frozen-thawed embryo transfer resulted in a higher birthweight than female sex and fresh embryo transfer which was also in agreement with other reports [31].

In our study, it is of interest that in the cleavage-stage cryopreservation group, embryos cultured in Vitrolife had significantly higher gestational age than those cultured in SAGE. The gestational age was 38.68 ± 1.34 weeks versus 38.39 ± 1.59 weeks, respectively. This result may be biased by the small sample size and the high elective cesarean rates. The cesarean rate in China was deeply influenced by the one child policy and other social factors. The cesarean rate was 75.8 % in SAGE group and 78.7 % in the other group.

As double embryo transfer is still the routine in China, vanishing twin effect [32] should be an indispensable phenomenon which has aroused a great interest these days. The prevalence rate of vanishing twin effect is among 10–12.5 % of IVF singletons [33, 34]. There is a great concern that the survivor of the twin will have a poorer prognosis than primary singleton which has been called “the black sheep of the family”. They were reported to suffer an increased risk of SGA, preterm birth, low birthweight and perinatal mortality [35, 36]. Furthermore, the gestational age at the time of vanishing was inversely correlated with adverse neonatal outcome [32]. To eliminate the possible bias from the vanishing effect, proportions of survivors as well as their vanishing time in different culture media were calculated and no statistical differences were found.

The limitations of our study were obvious. Firstly, it was a retrospective study. The maternal age between the two media in the fresh embryo transfer group were significantly different. However, our multiple regression analysis confirmed that it was not a confounder for neonatal birthweight in this study. Secondly, only two culture media were tested. Thirdly, the impact of culture media had not been studied in the context of blastocyst transfer. With respect to the controversial effects of prolonged in vrito cultrue (blastocyst transfer) on birthweight [37–40], it is necessary to collect data on neonatal outcomes from blastocyst transfer in the further study. Fourthly, the number of cases in two culture media varied a lot in this study. The reason was that SAGE was the major culture medium before 2012. However, since 2012, Vitrolife has gradually dominated the embryo culture media because it became the only medium to be permitted by Chinese Food and Drug Administration. The cases distribution was listed in detail (Table 5). However, these two culture media were used randomly during the study period and our embryo culture conditions as well as the technicians in our center remained the same. Last but not least, a follow up study is needed to explore the long term effects of culture media [41] and the potential epigenetic changes in human embryos that have been suspected lately [42].

**Table 5:** Cases distribution in two culture media

	Cleavage				Blastocyst
Year (s)	Vitrolife (ET cases)	SAGE (ET cases)	Vitrolife (FET cases)	SAGE (FET cases)	FET + ET (cases)
2009–2010	12	708	49	237	13
2011	138	430	78	135	81
2012	269	198	75	41	140

*ET* fresh embryo transfer

*FET* frozen-thawed embryo transfer

## Conclusion

In conclusion, commercially available types of culture media, Vitrolife and SAGE were not associated with birthweight of singleton newborns after fresh or frozen-thawed cleavage-stage embryo transfer.

## Abbreviations

ART: Assisted reproductive technology
GnRH: Gonadotropin-releasing hormone
HAS: Human serum albumin
hCG: Human chorionic gonadotropin
HRT: Hormone replacement therapy
ICSI: Intracytoplasmic sperm injection
IVF: In vitro fertilization
LGA: Large for gestational age
SGA: Small for gestational age
SSS: Synthetic serum supplement
